# The high prevalence of HPV and HPV16 European variants in cervical and anal samples of HIV-seropositive women with normal Pap test results

**DOI:** 10.1371/journal.pone.0176422

**Published:** 2017-04-20

**Authors:** Lays Paula Bondi Volpini, Neide Aparecida Tosato Boldrini, Luciana Bueno de Freitas, Angelica Espinosa Miranda, Liliana Cruz Spano

**Affiliations:** 1 Post-Graduate Program in Infectious Diseases, Federal University of Espírito Santo, Vitória, Brazil; 2 Department of Social Medicine, Center of Health Sciences, Federal University of Espírito Santo, Vitória, Brazil; 3 Department of Pathology, Center of Health Sciences, Federal University of Espírito Santo, Vitória, Brazil; Fondazione IRCCS Istituto Nazionale dei Tumori, ITALY

## Abstract

Human Immunodeficiency Virus (HIV)-seropositive women are more likely to have anogenital cancer, and high risk-HPV (HR-HPV) infection is the main associated factor. Between August 2013 and December 2015, we conducted a descriptive study to determine the HPV genotypes and HPV16 variants in cervical and anal samples of HIV-seropositive women with a normal Pap test. The viral DNA was amplified by PCR using the PGMY09/11 set of primers. Reverse line blot (RLB), restriction fragment length polymorphism (RFLP) and sequencing assays were used to determine the HPV genotypes. HPV16 variants were identified by gene sequencing. We found a high frequency of HR-HPV (60.3%; 76/126) at the anogenital site among HIV-seropositive women and without association with anal intercourse. HPV16 and European variant predominated among the HR-HPV. Mixed infections with at least three different HPV types were common, particularly at the anal site. CD4+ T-cell counts below 500 cells/mm^3^, a HIV viral load above 50 copies/mL and an age of 18 to 35 years old were all related to HPV anal infection. Our study showed a high frequency of HR-HPV in both cervical and anal sites of women with negative cytology belonging to a risk group for the development of anogenital cancer.

## Introduction

Human papillomavirus (HPV) is the most common sexually transmitted infection (STI) worldwide, and it is associated with development of anogenital cancers [1]. Over 200 different HPV genotypes have been identified [2]. Approximately 40 of them have tropism for the anogenital site. HPV strains are classified as low-risk (LR-HPV) and high-risk (HR-HPV) genotypes, according to risk of oncogenic progression [3]. Among HR-HPV strains, meta-analysis studies described HPV16 in approximately 50% of cervical cancers [4] and 70% of anal cancers [5]. Variations in the nucleotide sequence of this genotype classifies it as European (E) and non-European (NE) (African, Asian, Asian-American, North American), according to its geographical distribution. These variants differ in their biological properties and oncogenic potential, with a higher incidence of progression to malignancy conferred by the NE variants [6, 7].

Cervical cancers, the fourth most common cancer in women, are a serious problem of public health worldwide, with approximately 500,000 new cases annually, 85% of them in developing countries [8]. In turn, the incidence of anal cancer is rare in the general population (1/100,000), with an annual estimate of 27,000 new cases. However, a particularly high incidence occurs among men who have sex with men (MSM), women with a history of cervical cancer or vulvar cancer, and immunosuppressed populations [9]. Exposure to other STIs, especially HIV, increases the risk of both cervical cancer and anal cancer development [10]. According to Frisch et al. (2000) [11], HIV-infected women present a risk of 5.4-fold and 6.8 fold higher than HIV-seronegative women, for cervical and anal cancer development, respectively.

The control procedure for cervical cancer is mainly based on the detection of precursor lesions by cervical cytology, a practice adopted by many countries, and the World Health Organization (WHO) recommends cervical cancer screening every three years after a normal Pap test in HIV-seropositive women [12–14]. However, in developing countries, the incidence and mortality rates remain high [8], mainly due to screening coverage and methodological limitations [15]. On one hand, cytology has been associated with false negative results [16], and on the other, molecular screening test significantly reduces the mortality in cervical cancer cases, even in countries with limited resources [17]. Concerning anal cancer, there are no guidelines or randomized clinical trials to provide suitable screening of evidence-based recommendations [18]. The Brazilian Ministry of Health recommends annual Pap tests for HIV-seropositive women with receptive anal intercourse, HPV infection or cervical/vulvar abnormal histology [19].

In this context, molecular tests have emerged as useful tools for HPV genotyping beyond detection, providing an early indication of cervical and anal potential lesions, especially in HIV-seropositive women, who are at particularly high risk for HPV-related malignancies [12]. Therefore, this study aimed to probe the HPV genotypes and HPV16 variants in cervical and anal samples of HIV-seropositive women with a normal Pap test.

## Materials and methods

### Study design, population and ethical aspects

This study is a case series study in HIV-seropositive sexually active women, aged 18 to 65 years old who attended a STI/AIDS Reference Clinic in Vitoria, southeastern Brazil, from August 2013 to December 2015. Cervical and anal samples collected using a cytobrush were stored in Tris-EDTA buffer and maintained in a freezer at -70°C. A 20-minute face-to-face interview was conducted with the use of a standardized questionnaire, which included demographic (age, schooling, and marital status) and behavioural data (tobacco use, illicit drug use and history of anal intercourse). The CD4+ T-cell count and HIV viral load were measured in the Public Health Specialized Laboratory of Espírito Santo State, and the Pap test samples were probed in the Pathology Laboratory of Espírito Santo. Pap tests within the normal limits and benign cellular changes [20] were used as inclusion criteria and considered normal for the purpose of this study. Pregnancy was an exclusion criteria. This research obtained approval by the Ethical Research Council of the Center of Health Sciences of the Federal University of Espírito Santo, Brazil. All the participants signed an informed consent agreement.

### Laboratory procedures

DNA was obtained using the QIAamp DNA Mini Kit (Qiagen, Valencia, CA, USA) according to the manufacturer’s instructions. The HPV DNA was detected by amplification with the PGMY09/11 set of primers [21]. PCR for the *β-globin* [22] gene was performed in HPV negative samples as an extraction control and to assess the DNA integrity. All the HPV positive samples were genotyped using a reverse line blot (RLB) assay [23]. A restriction fragment length polymorphism (RFLP) [24] and sequencing assays using the BigDye Terminator v3.1 Kit (Applied Biosystems, Foster City, CA, USA) were used for samples that were not probed using the RLB assay.

The HPV genotypes identified in this study were classified as high-risk (HR-HPV 16, 18, 31, 33, 35, 39, 45, 51, 52, 56, 58 and 59), low-risk (LR-HPV 6, 11, 40, 42, 43, 44, 54, 61, 70, 72, 81 and 89) and undetermined-risk (UR-HPV HPV 2a, 3, 7, 10, 27, 28, 29, 30, 32, 34, 55, 57, 62, 67, 69, 71, 74, 77, 83, 84, 85, 86, 87, 90 and 91) [3]. The genotypes considered probable carcinogenic (26, 53, 66, 68, 73 and 82) [3] were grouped with HR-HPV.

Samples identified as HPV16 were subjected to PCR with primers for the HPV16 *LCR* region [25], which amplifies a common fragment of the variants of this genotype. To identify the specific nucleotide changes of each variant, sequencing was performed using the BigDye Terminator v3.1 kit (Applied Biosystems, Foster City, CA, USA). The sequences obtained were edited using BioEdit software v7.2.5 [26] and then aligned with the reference sequences of HPV16 for each sublineage [27] using the SeaView v4.5.4 program [28]. The construction of the phylogenetic tree of all the aligned sequences by the neighbour-joining method was conducted using the MEGA v6.0 software [29].

### Statistical analyses

The possible associations between the variables were tested using a chi-squared test with a Yates correction or Fisher's exact test, as appropriate. The HPV frequency of paired samples (cervical and anal) was compared using the McNemar test. The Odds Ratio (OR) and confidence intervals were calculated using bivariate analysis to estimate the degree of association between infection and demographic variables. Variables were considered significant when the p value was < 0.05.

## Results

### Samples

In total, 126 HIV-seropositive women with a normal Pap test participated in this study. Demographic, behavioural and clinical data of the study population are shown in Table 1. The average age of the women was 41.4 years old (SD ± 10.3), and the average length of schooling was 8.4 years (SD ± 3.7).

**Table 1:** Demographic, clinical and behavioural characteristics of HIV-seropositive women who attended the STI/AIDS Reference Clinic in Vitória from August 2013 to December 2015.

Characteristics^a^	n (%)
**Demographic**	
Marital status (n = 124)	
Married	66 (53.2)
Single	38 (30.6)
Divorced/widow	20 (16.2)
**Clinic**	
HAART^b^ use (n = 125)	102 (81.6)
HIV Viral load, copies/mL (n = 109)	
<50	70 (64.2)
50–400	7 (6.4)
>400	32 (29.4)
CD4+ count, /mm^3^ (n = 109)	
<200	6 (5.5)
200–500	32 (29.4)
>500	71 (65.1)
**Behavioral**	
Smokers (n = 122)	23 (18.9)
Receptive anal intercourse (n = 120)	82 (68.3)
Illicit drug (n = 120)	30 (25)

^a^Due to the lack of data for some variables in the study, the sample size varied for each analysis.

^b^HAART—Highly active antiretroviral therapy.

### Detection and HPV genotyping

HPV DNA was observed in 71.4% (90/126) of women, 38.9% (49/126) in the cervical site and 60.3% (76/126) in the anal site (p = 0.001); cervical and anal infection was concomitant in 38.9% (35/90) of the positive cases. The presence of amplifiable DNA was confirmed by PCR of the *β-globin* gene in all HPV negative samples. HR-HPV occurred in 60.3% (76/126) of the cervical and anal samples of the women, with a higher frequency at the anal site of 46.8% vs. 32.5% (p = 0.0284).

**Fig 1:**
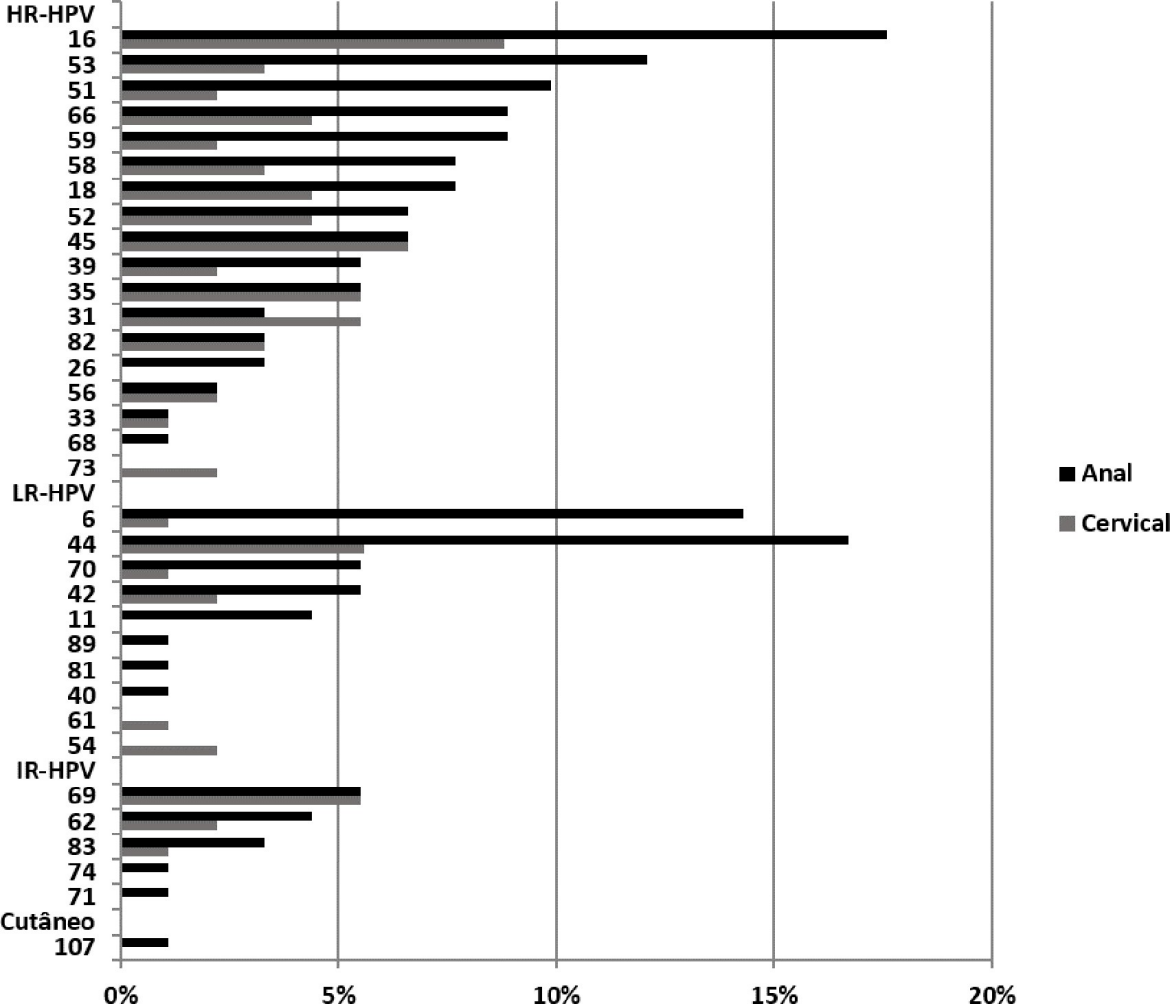
The frequency of HPV genotypes. The frequency of HPV genotypes at the cervical and anal sites of HIV-seropositive women with a normal Pap test. X axis: frequency of positive cases for each HPV genotype; Y axis: HPV genotypes (classified by risk).

Overall, 34 distinct HPV genotypes were detected in both samples in the study population (Fig 1). HPV16 was the most prevalent type at the cervical and anal sites (8.9% and 17.8%, respectively), followed by types 45 (6.7%), 31, 35, 69, 44 (5.6% each), 18, 52, and 66 (4.4% each) at the cervical site and types 44 (16.7%), 6 (14.4%) and 53 (12.2%) at the anal site (Fig 1). Among the cases of concomitant cervical and anal infection, at least one concordant genotype occurred in 48.6% (17/35).

At least three distinct types of all HPV occurred concomitantly in 78.6% of anal samples and in 21.4% of cervical samples (OR = 2.9, 95% CI 1.09 to 7.83; p = 0.03) (Table 2). No more than four or seven types were observed at the cervical and anal sites, respectively.

**Table 2:** The distribution of multiple infections at the cervical and anal sites of HPV positive women (n = 90).

# of types	Cervical n (%)	Anal n (%)	Total n (%)
**≤ 2 types**	43 (44.3)	54 (55.7)	97 (100)
**≥ 3 types**^a^	6 (21.4)	22 (78.6)	28 (100)
**Total**	49	76	125

^a^p = 0.03

### HPV16 variants

**Fig 2:**
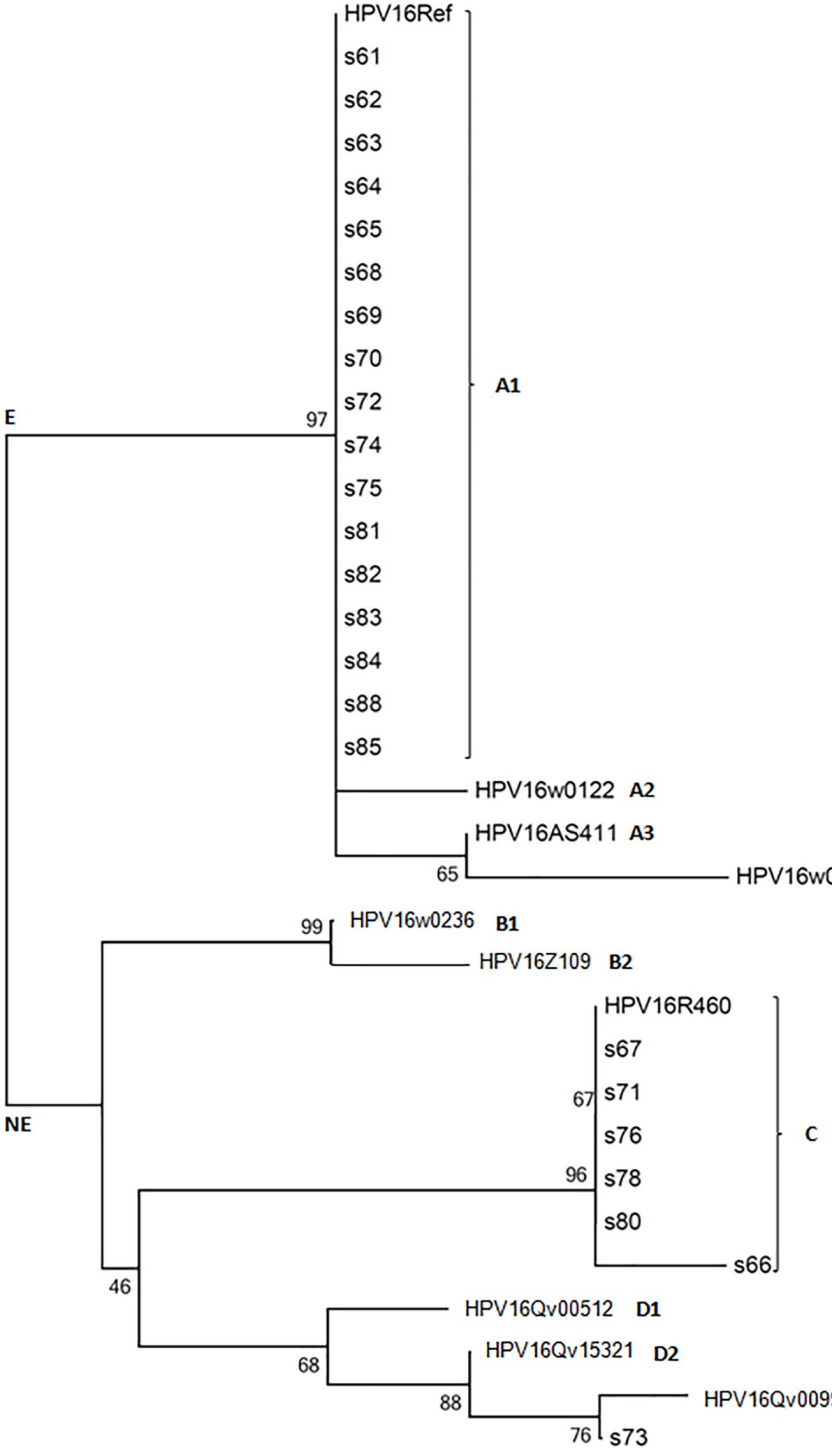
Tree topology. The phylogenetic tree created using the neighbour-joining method from the global alignment of full and partial sequences of the HPV16 genome. E-European variants (sublineages A1-A4); NE-non-European variants (sublineages B1-D3). The reference sequences of each HPV16 lineage was obtained from GenBank (ID/lineage/acession number: 16Ref/A1/K02718; w0122/A2/AF536179; AS411/A3/HQ644236; w0724/A4/AF534061; w0236/B1/AF536180; Z109/B2/HQ644298; R460/C/AF472509; Qv00512/D1/HQ644257; Qv15321/D2/AY686579; Qv00995/D3/AF402678).

Twenty-four samples from 20 women had the HPV16 partial genome (*LCR* region) characterized: four from both cervical and anal sites, 12 from solely the anal site and four from solely the cervical site. The nucleotide sequences obtained were compared with the HPV16 prototype of each HPV16 variant lineage and sublineage, and each one was assigned to a specific lineage based on the phylogeny (Fig 2). Phylogenetic analysis classified 70.8% (17/24) of the samples as HPV16 European (E, A lineage) and 29.2% (7/24) as non-European variants (NE, lineages B, C, and D). The non-European variants were represented by six African 2a (lineages C) variants and one Asian American (lineages D3) variant. The four cases of concomitant infection showed the same variants between both anatomical sites (3 European and 1 African 2a).

### Factors associated with cervical and anal HPV

The presence of cervical HPV was a factor associated with the presence of anal HPV, with 71.4% (35/49) of positive cases of cervical HPV also present at the anal site (OR = 2.92; 95% CI = 1.56–5.81; p< 0.01) (Table 3).

**Table 3:** The frequency of positive and negative cases of HPV at the cervical and anal sites of HIV-seropositive women with normal Pap test results who attended the STI/AIDS Reference Clinic in Vitória from August 2013 to December 2015.

Cervical HPV	Anal HPV n (%)		Total
	Positive	Negative	
**Positive**	35 (71.4)	14 (28.6)	49 (100)
**Negative**	41 (53.2)	36 (46.8)	77 (100)
**Total**	76	50	126

Among the variables investigated with HPV occurrence, anal infection was significantly associated with women younger than 35 years (p = 0.01), a CD4+ T-cell count below 500 cells/mm^3^ (p = 0.01) and a HIV viral load above 50 copies/mL (p = 0.001) (Table 4).

**Table 4:** The odds ratio (OR) and confidence interval of HPV infections that were exclusively cervical, anal, or concomitant cervical and anal, according to the demographic, clinical, and behavioural characteristics of HIV-seropositive women with normal Pap test results obtained by the STI/AIDS Reference Clinic in Vitória from August 2013 to December 2015.

Characteristics^a^	Cervical (n = 126)				Anal (n = 126)				Anal/Cervical (n = 90)			
Age (years)	Pos	Neg	OR (95% IC)	p	Pos	Neg	OR (95% IC)	p	Pos	Neg	OR (95% IC)	p
<35	13 (43%)	17 (57%)	0.78 (0.34–1.80)	0.6	24 (80%)	6 (20%)	0.29 (0.11–0.79)	0.01	13 (54%)	11 (46%)	2.36 (0.91–6.12)	0.07
>35	36 (38%)	60 (62%)			52 (54%)	44 (46%)			22 (33%)	44 (67%)		
**CD4+count (cells/mm**^**3**^**)**												
<500	19 (50%)	19 (50%)	0.45 (0.20–1.01)	0.05	29 (76%)	9 (24%)	0.34 (0.14–0.81)	0.01	16 (50%)	16 (50%)	2.54 (0.99–6.53)	0.05
≥500	22 (31%)	49 (69%)			37 (52%)	34 (48%)			13 (28%)	33 (72%)		
**HIV Viral load (copies/mL)**												
<50	24 (24%)	46 (66%)	1.48 (0.66–3.30)	0.3	34 (49%)	36 (51%)	4.8 (1.88–12.44)	0.001	13 (29%)	32 (71%)	0.43 (0.69–1.10)	0.07
≥50	17 (44)	22 (56%)			32 (82%)	7 (18%)			16 (48%)	17 (52%)		
**Smoking**												
Yes	9 (39%)	14 (61%)	0.93 (0.37–2.35)	0.9	12 (52%)	11 (48%)	1.54 (0.62–3.83)	0.3	7 (50%)	7 (50%)	1.80 (0.57–5.72)	0.3
No	37 (37%)	62 (62%)			62 (63%)	37 (37%)			26 (36%)	47 (64%)		
**Drugs use**												
Yes	14 (47%)	16 (53%)	0.63 (0.27–1.46)	0.3	19 (63%)	11 (37%)	0.91 (0.39–2.14)	0.8	12 (57%)	9 (43%)	2.49 (0.92–6.79)	0.06
No	32 (36%)	58 (64%)			55 (61%)	35 (39%)			23 (35%)	43 (65%)		
**Receptive anal intercourse**												
Yes	32 (39%)	50 (61%)	0.91 (0.41–2.01)	0.8	52 (63%)	30 (37%)	0.79 (0.36–1.74)	0.6	25 (42%)	34 (58%)	1.84 (0.70–4.84)	0.2
No	14 (37%)	24 (63%)			22 (58%)	16 (42%)			8 (29%)	20 (71%)		
**Schooling (years)**												
≤ 5	11 (37%)	19 (63%)	1.03 (0.44–2.42)	0.9	15 (50%)	15 (50%)	1.76 (0.76–4.04)	0.2	8 (44%)	10 (56%)	1.47 (0.51–4.21)	0.5
>5	34 (37%)	57 (63%)			58 (64%)	33 (36%)			24 (35%)	44 (65%)		

^a^The number varies due to the lack of data for some variables.

## Discussion

The HPV test, a useful tool for monitoring women with equivocal or normal cytological results [30, 31], was applied in this study to investigate HR-HPV in HIV-seropositive women with normal cervical Pap tests. We first demonstrated a high prevalence of HPV infection in this population, similar to the overall prevalence observed by Clifford et al. (2006) [32] in a meta-analysis evaluating the HPV types in HIV-seropositive women with and without cervical lesions. In the comparison between cervical and anal HPV infection, we showed greater frequency at the anal site, which was also previously reported by Palefsky et al. (2001) [33] (79% vs. 53%) in both HIV-seropositive and HIV-seronegative women, although less frequently in the latter (43% vs. 24%). These data suggest that there may be a greater persistence in HPV infection or in replication rate at the anal site than at the cervical site, which can be a source of infection for the female genital tract.

The great diversity of HPV types was shown in our study using the RLB methodology complemented by the RFLP method and sequencing, which was also shown by Gonçalves et al. (2008) [34] and Kojic et al. (2011) [35] in a similar population using RFLP and linear array as typing methods. The cutaneous type HPV107 (*Betapapillomavirus* genus) we observed in the genital samples suggests possible transmission during sexual contact. This finding is not exactly unexpected since Torres et al. (2015) [36] found HPV107 among the most prevalent cutaneous types (β and γ-*papillomavirus*) at the anal site of HIV-positive MSM.

We highlighted the high frequency of HR-HPV at both cervical and anal sites, which was significant in the latter site, similar to previous reports [34, 35]. This result is worrying because it is in relation to HIV-seropositive women with normal Pap test and the inherent risk for high-grade lesion development, as noted by Keller et al. (2015), who observed a 5-year cumulative risk for CIN-2+ in 16% of the cytological cases that were previously normal [37]. HPV16 was the most prevalent type at both cervix and anal sites, which is in contrast with other studies [34, 35, 38]. Once again, this result has important implications regarding the risk of anal cancer and also the 3-year risk of CIN-2+ of 29% and 13-fold greater risk of CIN-3+ compared to those not infected with HR-HPV [37].

In a multicentric study, Cornet et al. (2013) [39] identified the European variant as the most common in all regions of the world, except in sub-Saharan Africa and East Asia, while the African variant predominated the northern sub-Saharan region of Africa and the Asian variant in East Asia. Indeed, the worldwide distribution of variants varies according to the geographical area and correlates with the level of intrinsic admixture of each population and recent patterns of human migration [40, 41]. In our country, the European HPV16 variant predominates, as shown by our analysis in this study and by others’ studies [40, 42]. These results are, in part, a consequence of the European colonization of Brazil, the African slave trade during the 16^th^ to late 19^th^ century and Asiatic immigration in the 20^th^ century. Although all HPV16 variants are related to the development of precancerous lesions and cancer, the predominance of European variants in this study implies a lower risk for the development of HSIL [40, 42].

Infection with multiple HPV types was greater at the anal site in relation to the cervical site. It is unclear whether this fact could increase the pathogenesis of intraepithelial lesions as previously suggested [33]. However, multiple HPV infections at the anal site would be associated with the development of HSIL at this site [43] or even with an increased risk of subsequent infection in adjacent anatomical sites [44]. Indeed, we demonstrated that half of the cervical-anal concomitant infections was by the same HPV genotype. Likewise, the cervix could act as a source for anal HPV infection, which is enhanced by the observation that hygiene habits would facilitate the transmission to the anus and the contamination of the perineum with vaginal discharge could happen [45]. Our results also showed that cervical HPV can be considered an associated factor with anal HPV infection (OR = 2.2) and anal intercourse was not statistically associated with the presence of HPV at the anal site, similar to previously observed results [33, 35]. Therefore, we are seeing a population of women without cervical cytological abnormalities, some of them without history of anal intercourse, which is not included in the anal cancer screening proposed by the Brazilian Ministry of Health for HIV-seropositive women who recommends anal Pap tests for women with a history of receptive anal sex, HPV infection or abnormal cervical or vulvar histology [19]. These recommendations, therefore, ignore the high prevalence of HR-HPV and the inherent risk for high grade lesions in these women beyond the dissociation between anal infection and anal intercourse. The presence of HPV was significant at the anal site, especially in women younger than 35 years, similar to the results described by Palefsky et al. (2001) [33]. Factors related to HIV infection, such as low CD4+ T-cell counts (<500 cells/mm^3^) and detectable viral load (>50 copies/mL), were also statistically associated with the presence of HPV at the anal site. Indeed, previous studies have demonstrated that a lower CD4+ count is associated with anal HPV [33, 35] and detectable HIV viral load, with abnormal anal cytology [46].

Although a descriptive study is not the best model to determine risk factors, it was not our main objective, and its application may be justified to assess the prevalence of factors associated with HPV infection at the cervical and anal sites of HIV-seropositive women.

The value of the implementation of molecular tests in cervical cancer screening for HIV-seropositive women has been emphasized [37], and the cost-benefit analysis for developing countries supports the implementation of molecular tests in cervical cancer screening [47]. Since that our study showed a high frequency of HR-HPV in both cervical and anal sites of women with negative cytology belonging to a risk group for the development of anogenital cancer, molecular tests could be an additional tool to improve the follow up of this population.
