# Correction: The high prevalence of HPV and HPV16 European variants in cervical and anal samples of HIV-seropositive women with normal Pap test results

**DOI:** 10.1371/journal.pone.0178357

**Published:** 2017-05-18

**Authors:** The PLOS ONE Staff

The image for Fig 2 is incomplete. Please see the complete, correct Fig 2 here. The publisher apologizes for the error.

**Fig 2:**
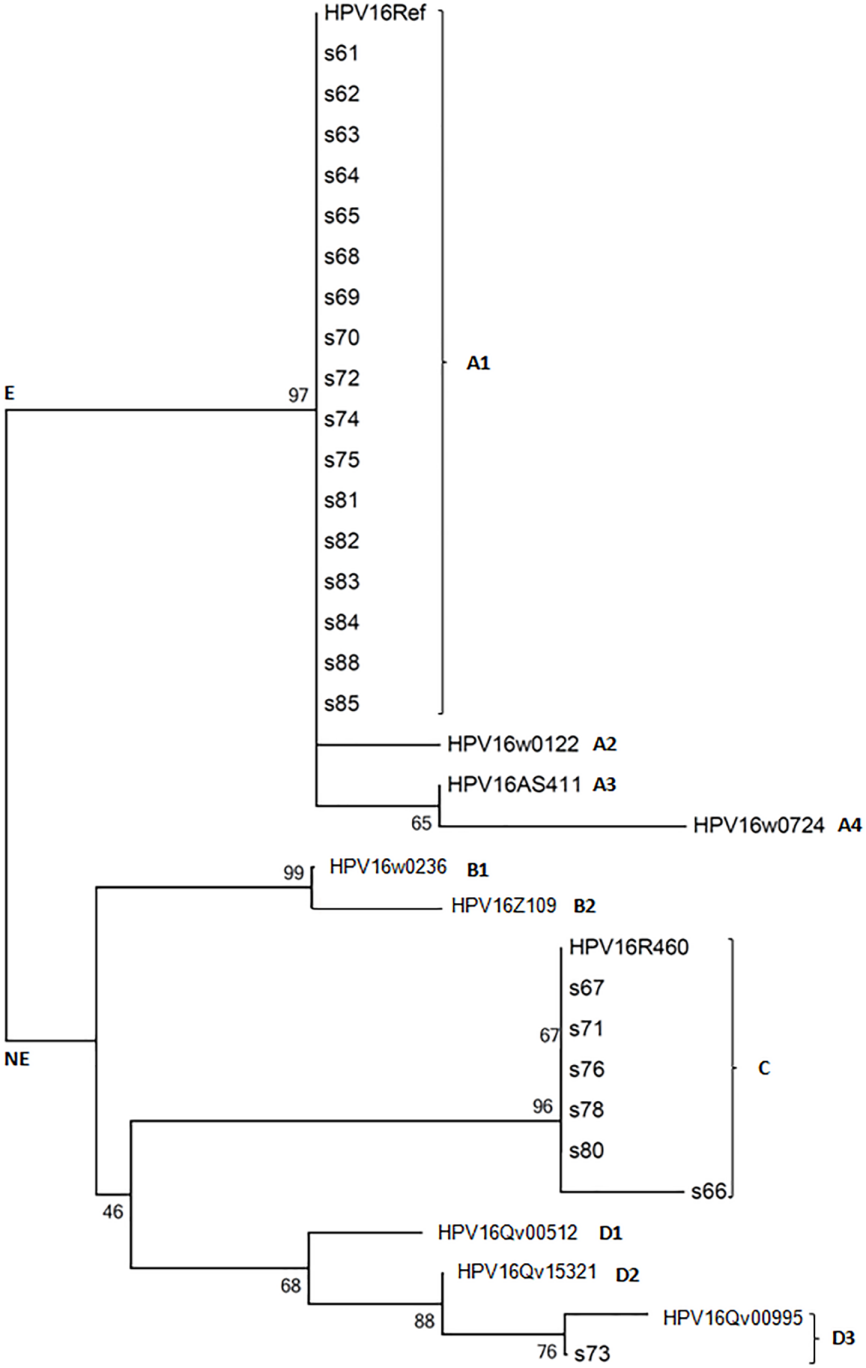
Tree topology. The phylogenetic tree created using the neighbour-joining method from the global alignment of full and partial sequences of the HPV16 genome. E-European variants (sublineages A1-A4); NE-non-European variants (sublineages B1-D3). The reference sequences of each HPV16 lineage was obtained from GenBank (ID/lineage/acession number: 16Ref/A1/K02718; w0122/A2/AF536179; AS411/A3/HQ644236; w0724/A4/AF534061; w0236/B1/AF536180; Z109/B2/HQ644298; R460/C/AF472509; Qv00512/D1/HQ644257; Qv15321/D2/AY686579; Qv00995/D3/AF402678).
